# Supplementary material for: Topological radiation from vortex masers
Source: Nat Commun. 2025 Nov 28;16:10750. doi: 10.1038/s41467-025-65787-9 (PMC12663456; doi:10.1038/s41467-025-65787-9)
Supplement: Supplementary file 1 — Supplementary Information [file 41467_2025_65787_MOESM1_ESM.docx]

Supplementary Information for

Topological radiation from vortex masers

Haoye Qin^1,#^, Rongrong Xiang^1,#^, Amir Jafargholi^1^, Zhe Zhang^1^, Romain Fleury^1†^

*^1^Laboratory of Wave Engineering, École Polytechnique Fédérale de Lausanne,*

*1015 Lausanne, Switzerland*

*^#^These authors contributed equally: Haoye Qin, Rongrong Xiang*

*Corresponding author: ^†^romain.fleury@epfl.ch*

**Supplementary Figures**

*
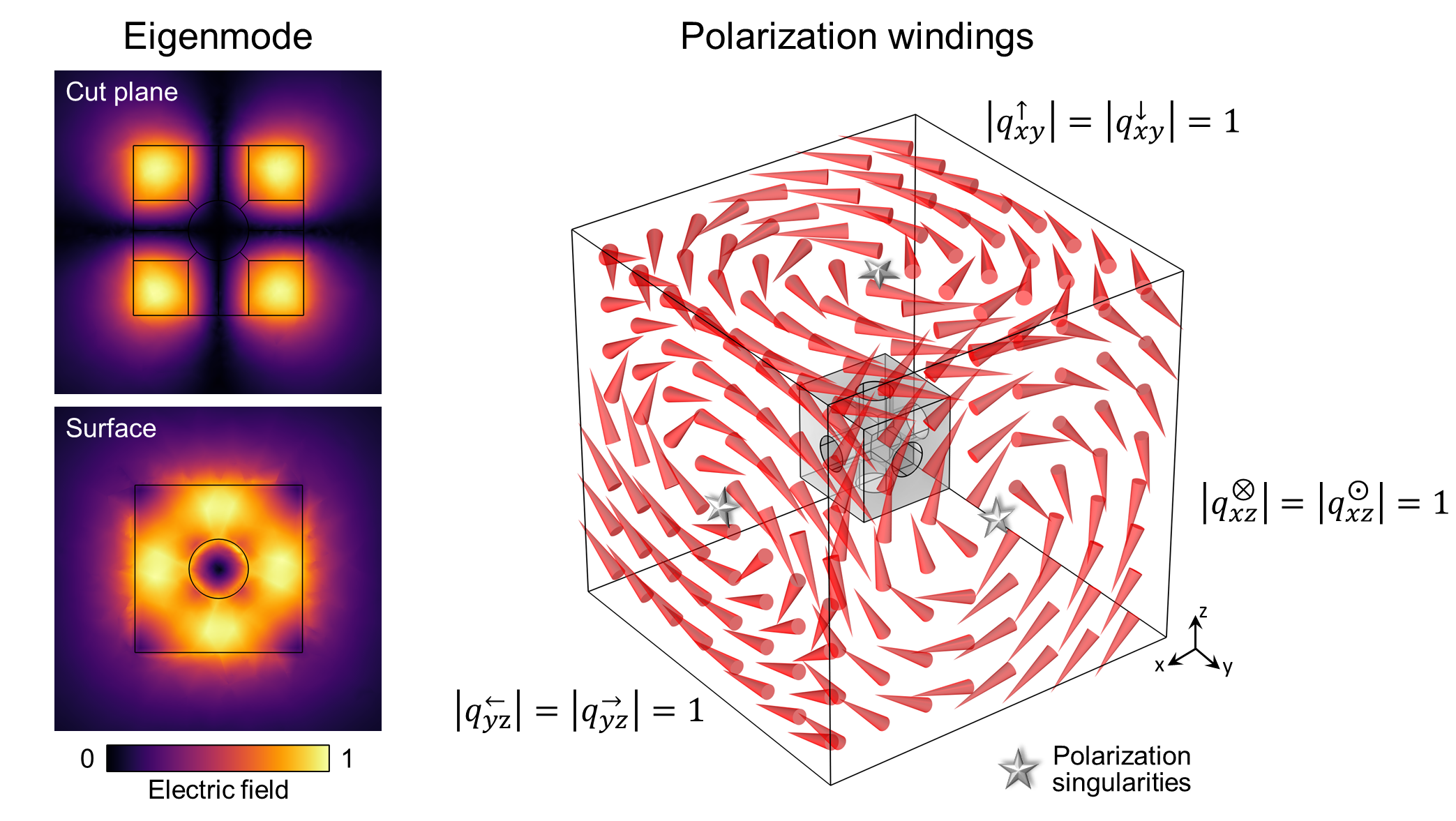
*

**Supplementary Fig. S1: Illustration of multiple topological polarization singularities induced by the cubic cavity eigenmode**. Left: electric eigenmode of the cubic cavity at the central cut plane and outer surface. Right: vectorial electric field distribution on planes parallel to each surface of the STO cubic cavity. Multidimensional topological singularities can be retrieved from the topological charge above each surface as the winding charge of electric field polarization angle (or orientation angle), $q_{mn}^{k}=\frac{1}{2\pi}\oint_{C} dr\cdot\nabla_{r}\theta(r)$, where *θ* (*r*) is the position *r* dependent polarization angle on the *mn* plane, *C* is a closed loop surrounding the plane center, and $k\in\{\uparrow,\downarrow,\leftarrow,\to,\bigotimes,⨀\}$ indicates the radiation direction.

*
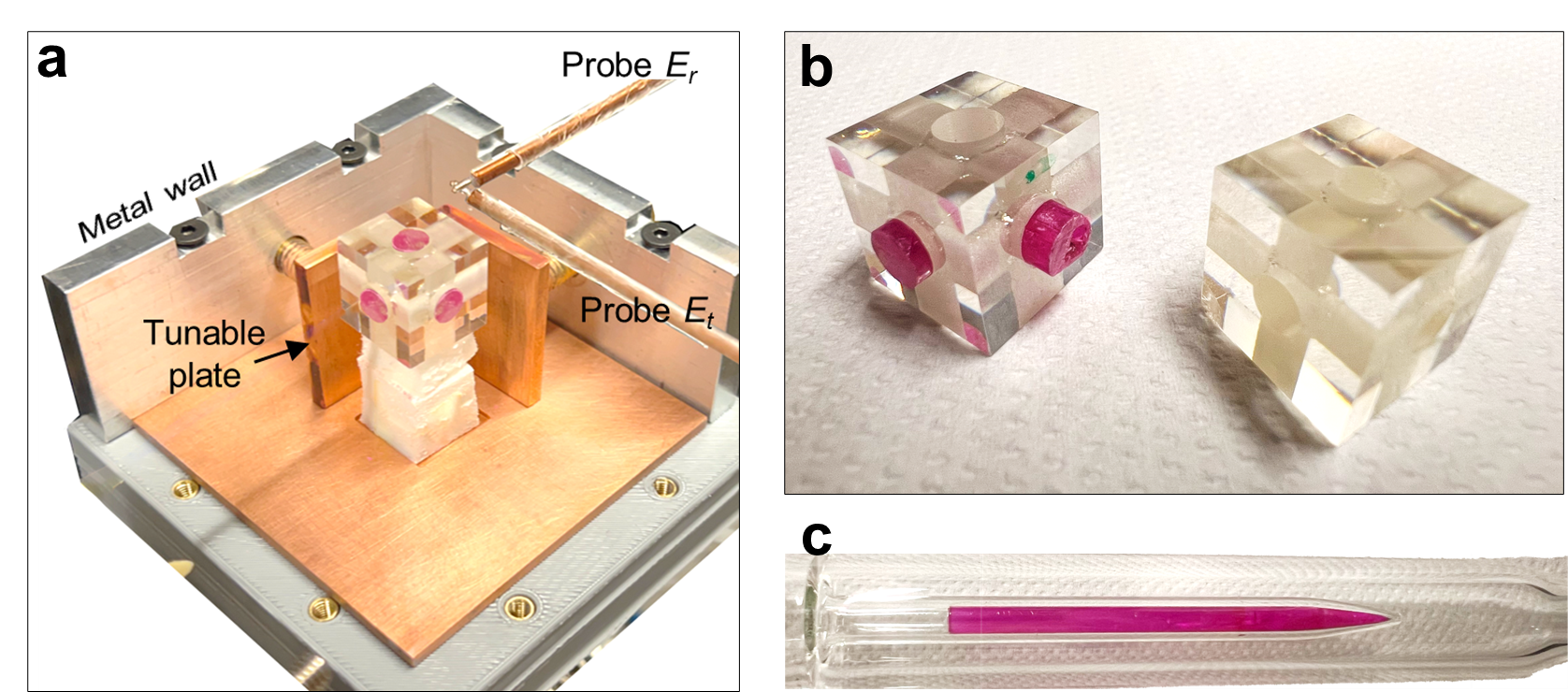
*

**Supplementary Fig. S2: Measurement setup of 3D vortex maser. a,** Configuration of the vortex maser experiment setup with two-sidewall metal holder and two movable plates for fine-tuning resonance of cubic cavity, enabling masing vortices in three dimensions and related measurement. Two electric probes are denoted as probe *E_r_* and *E_t_* for radial and tangential direction. **b,** Photo of two polished STO cubes with three symmetric through holes. **c,** Growth of pentacene-doped p-terphenyl crystal.

*
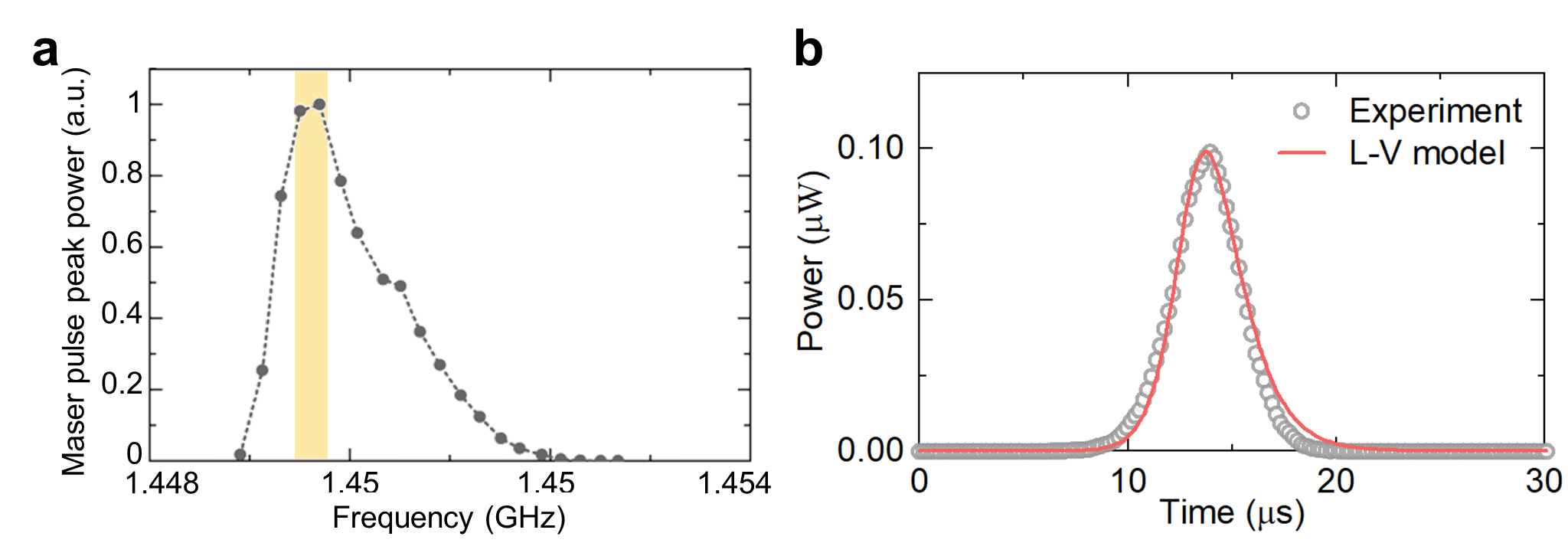
*

**Supplementary Fig. S3: Gain medium profile and modeling of masing pulse. a,** Gain linewidth of the Pentacene: p-terphenyl crystal and resonance frequency of the cubic cavity (shaded region). The cavity’s resonance can be fine-tuned through temperature and distances of metal walls. **b,** Output power profile (gray circles) extracted from the pulses along with a fitted curve using the Lokta-Volterra model (L-V model, blue line).

*
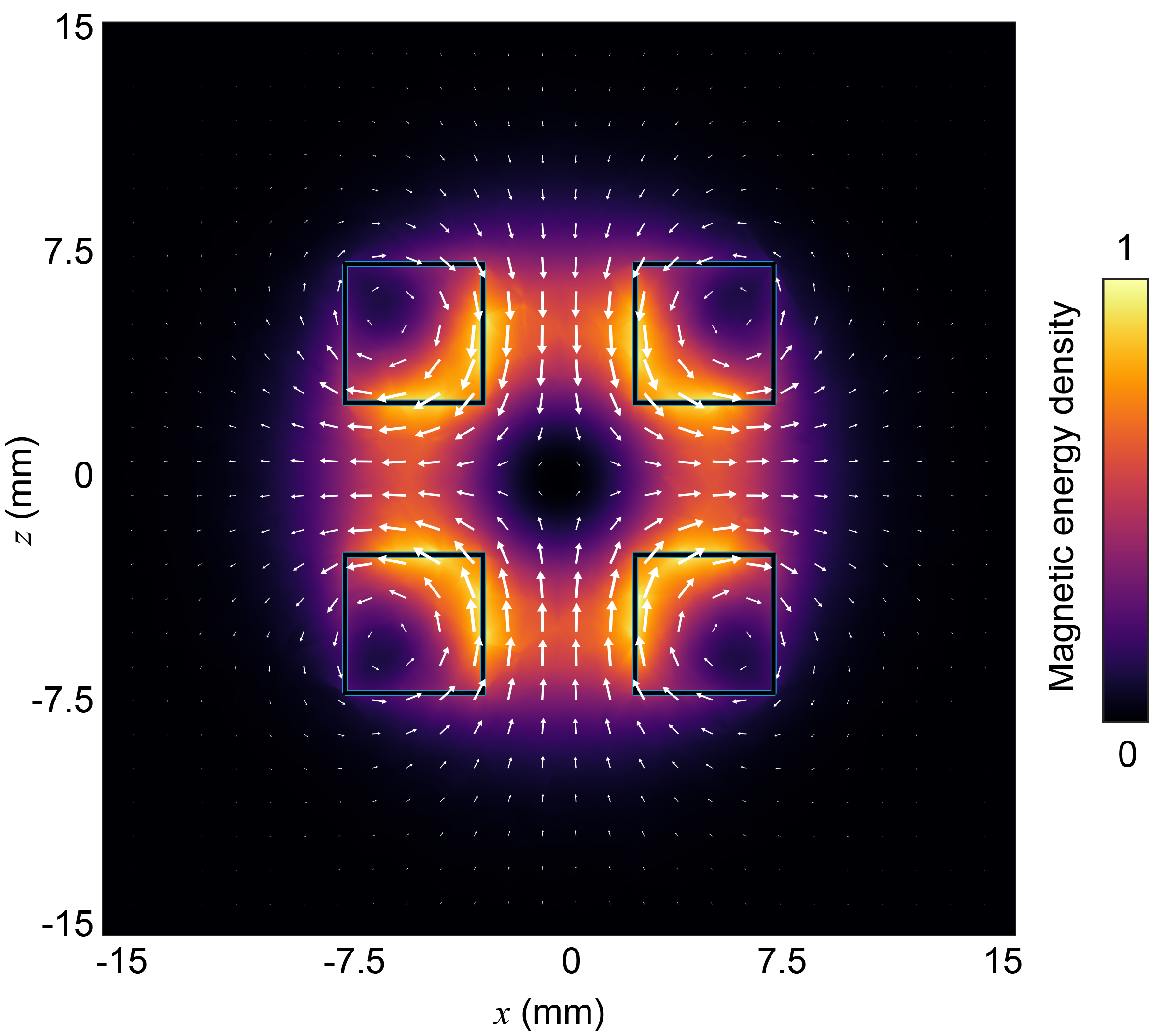
*

**Supplementary Fig. S4: Localized magnetic energy distribution.** 2D cut-plane of the magnetic energy density in the cubic cavity showing strong localization within the air hole.

***
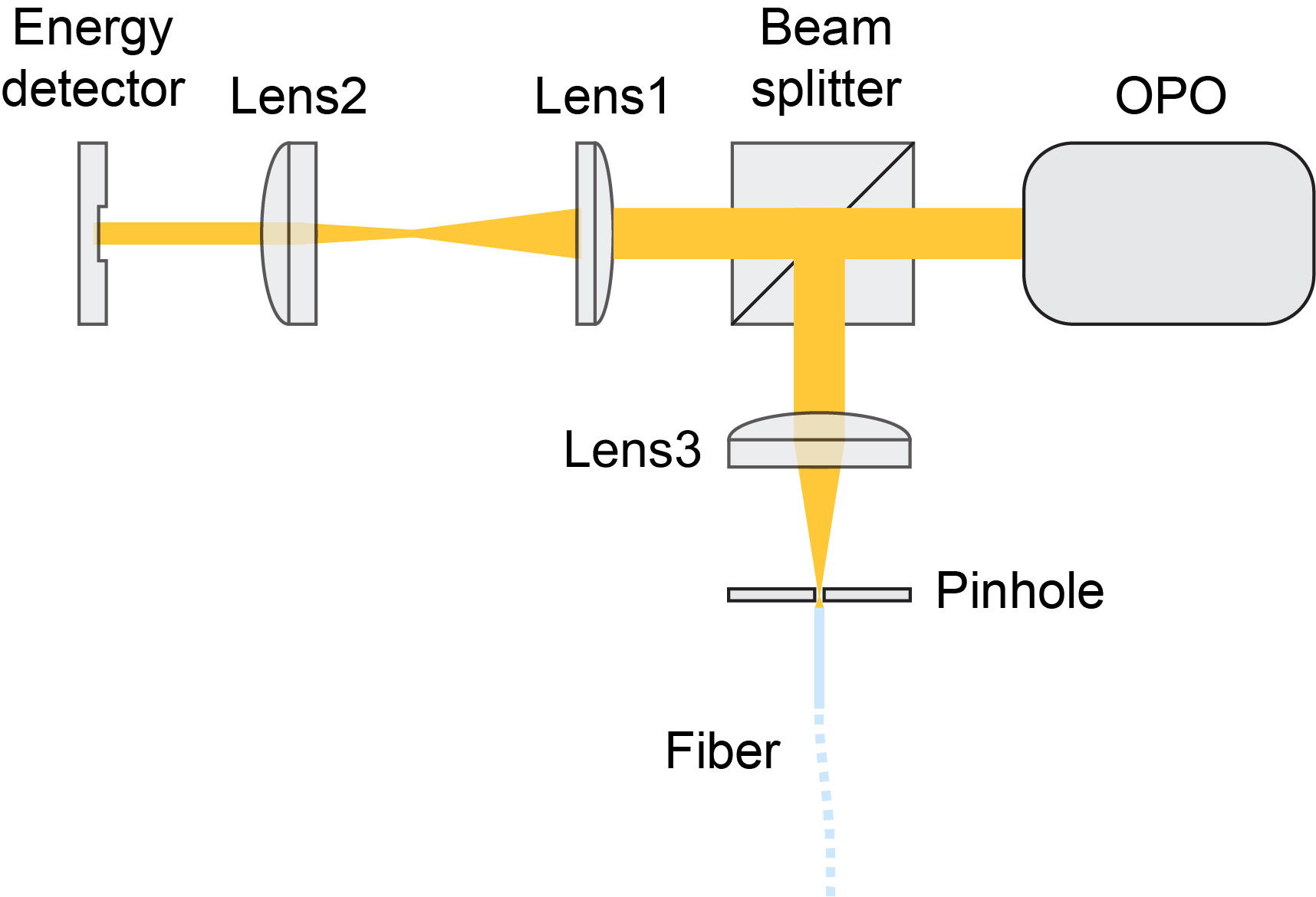
***

**Supplementary Fig. S5: Experimental configuration of optical excitation.** Optical path for coupling the optical parametric oscillator (OPO) output into a multi-mode optical fiber to pump the gain medium in the maser.

*
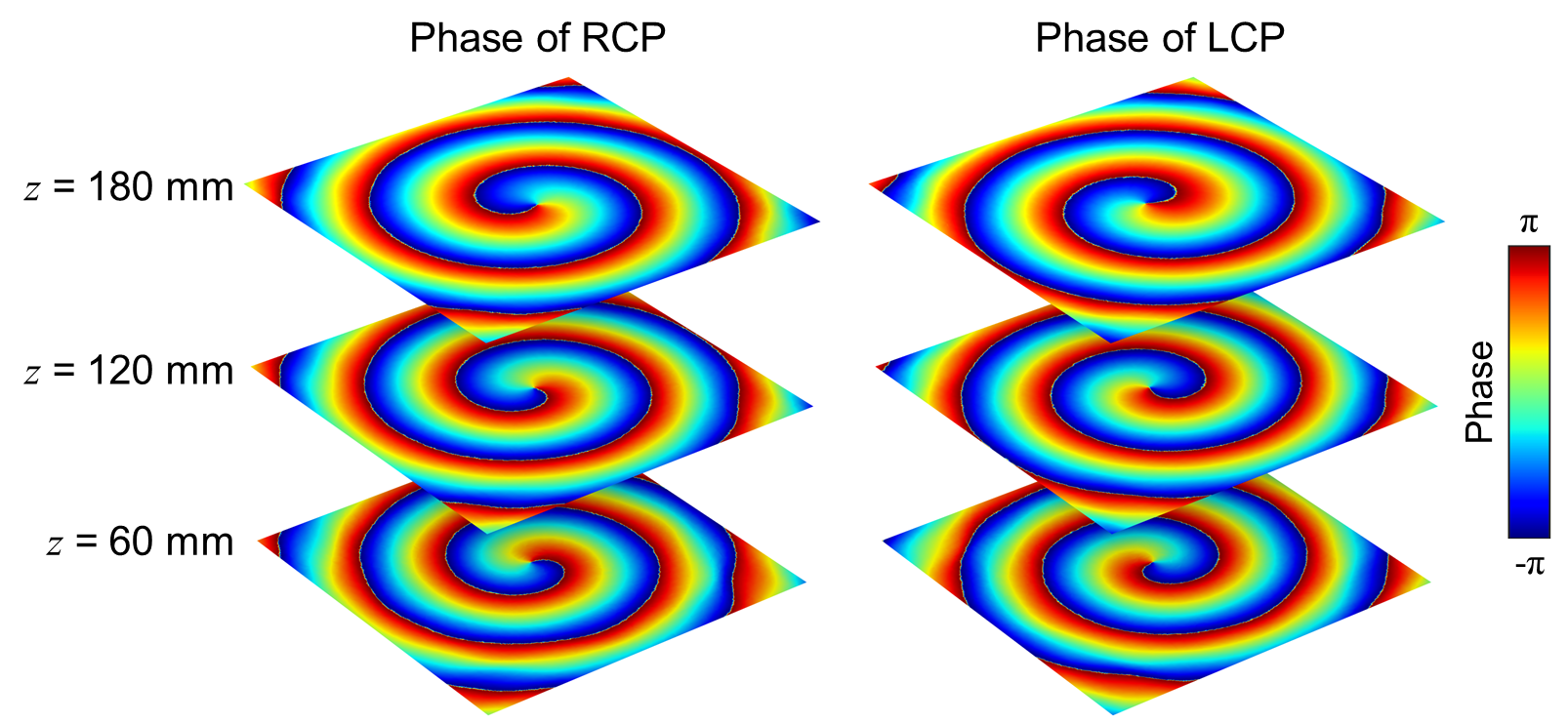
*

**Supplementary Fig. S6: Simulated phase distribution for two spins above the maser cube.** Phase distribution projected to two circular polarizations is plotted at *z* distance of 60 mm, 120 mm, and 180 mm above the cube.

*
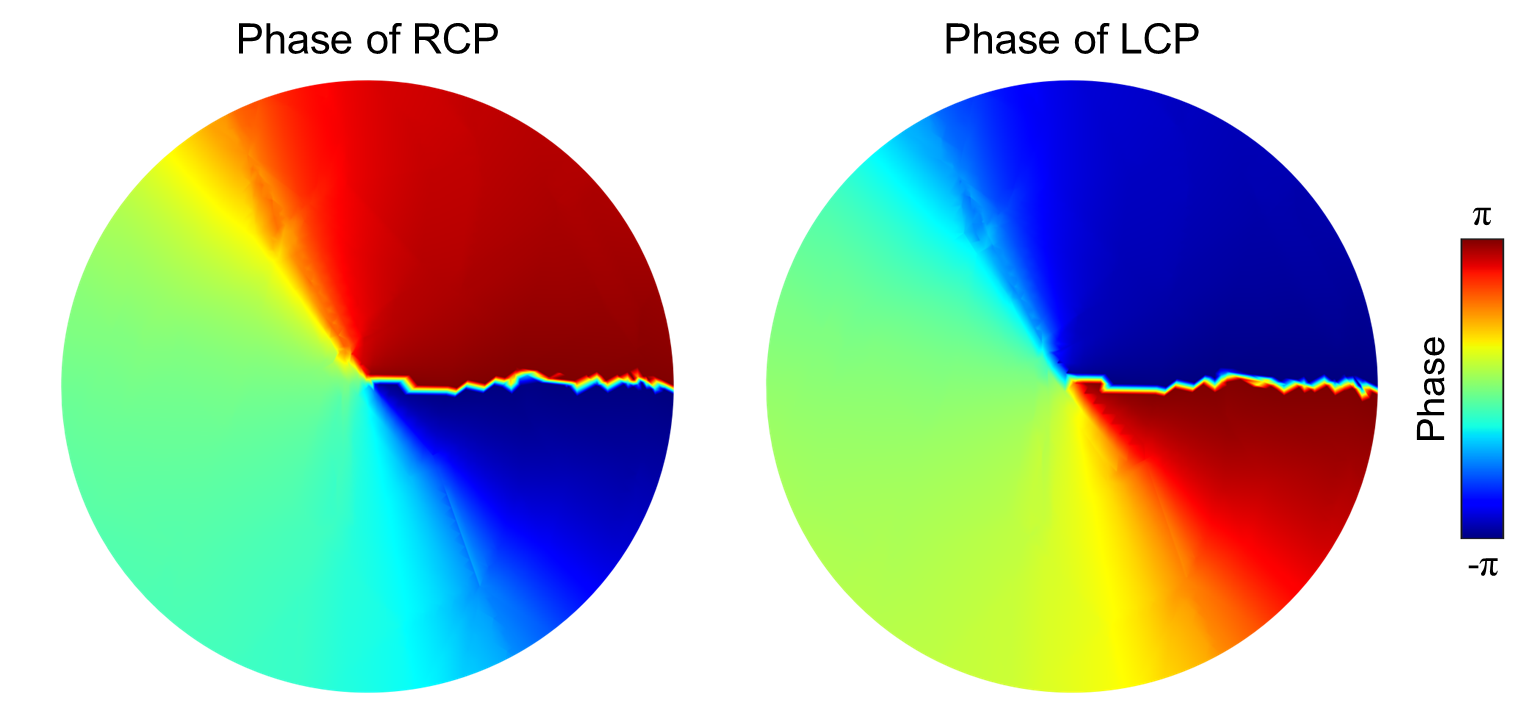
*

**Supplementary Fig. S7: Simulated OAM phase above the metasurfaces.** Two kinds of metasurfaces are placed above the cubic cavity and the phase profiles of RCP and LCP are extracted, verifying that the metasurface induces a selection of maser pulses with positive or negative angular momentum.


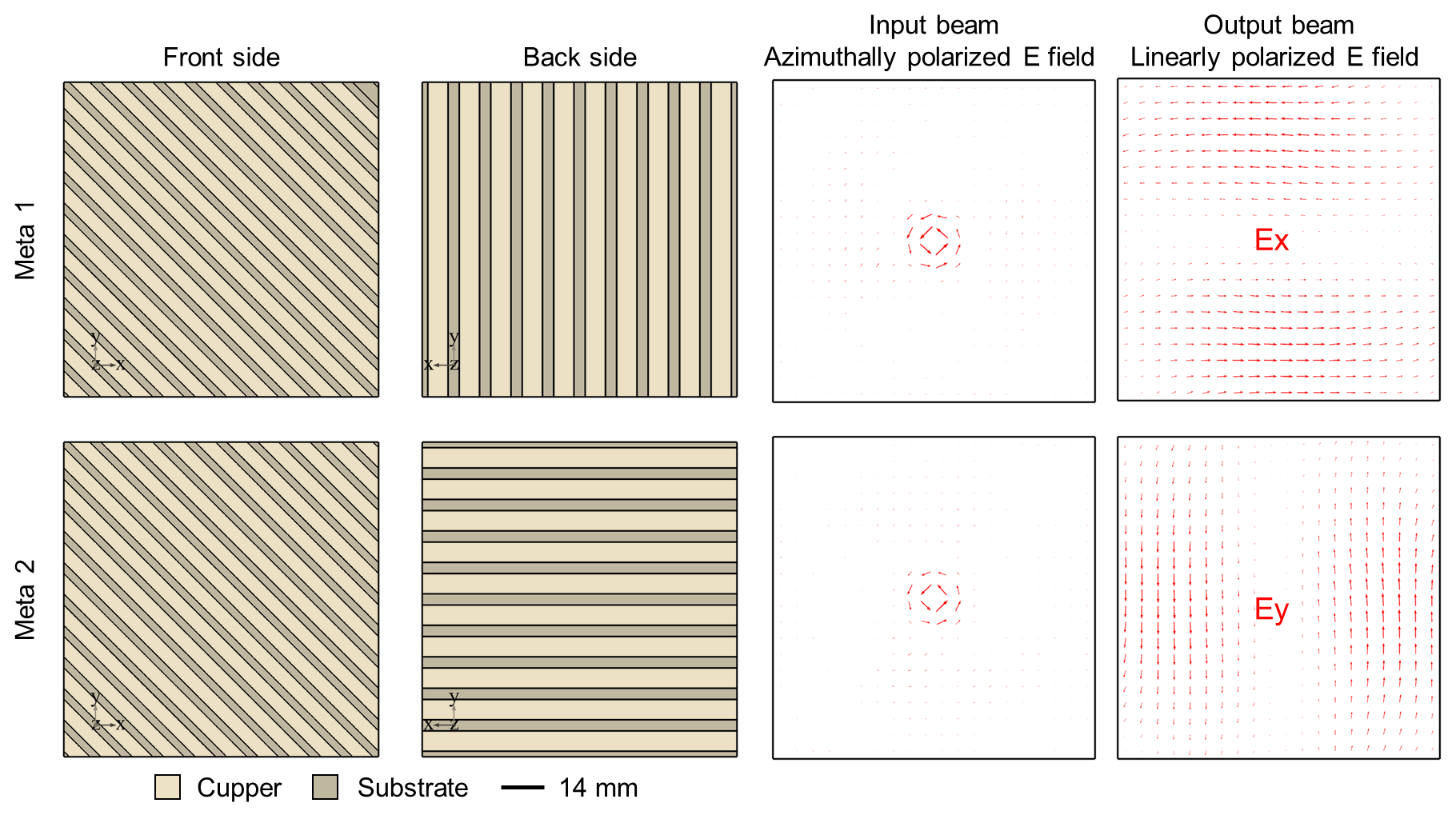


**Supplementary Fig. S8: Designed layout and simulated performance of spin-decoupling metasurfaces.** Metasurfaces are equipped with double-sided metal strips. The metal strips have a width of 7 mm and a gap of 3.8 mm. The metasurface blocks RCP (LCP) beam and lets LCP (RCP) beam pass, resulting in the OAM of unit charge.


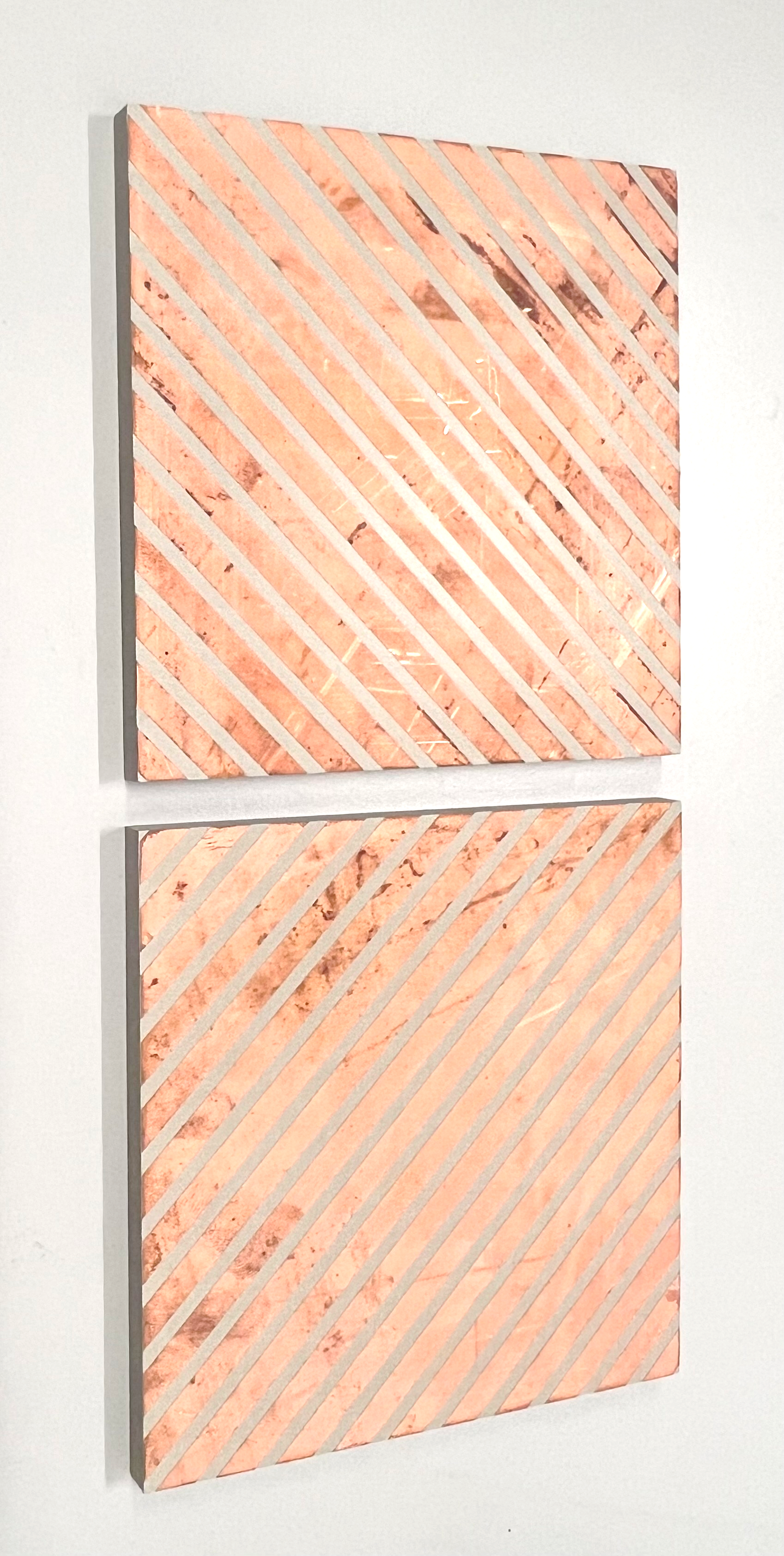


**Supplementary Fig. S9: Fabricated metasurfaces with grating on dielectric substrates.** Two metasurfaces featuring double-faced metal gratings are fabricated for the purposes of spin decoupling and detection of OAM masing.

***
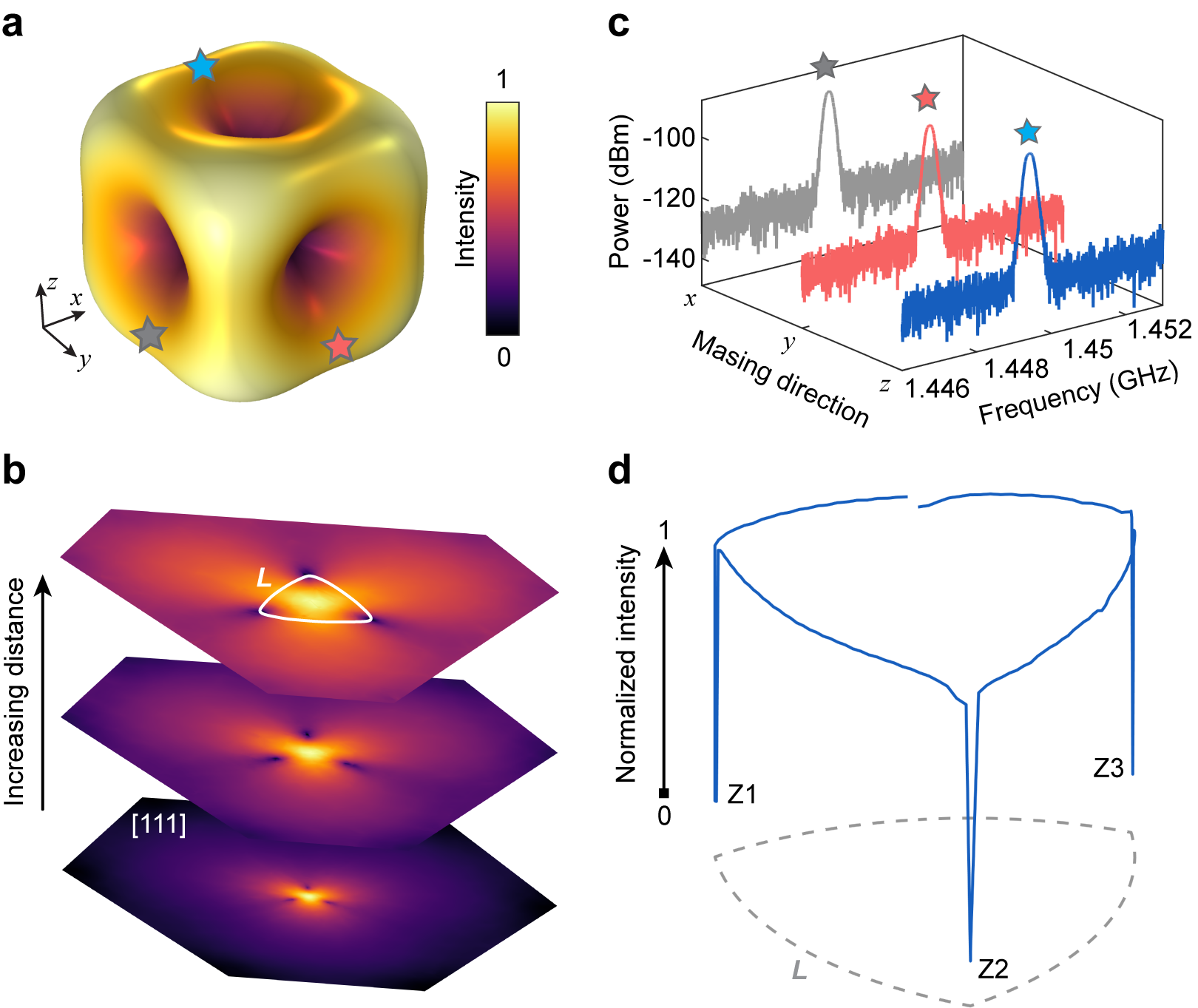
***

**Supplementary Fig. S10: Coexistence of 3D masing and singularities.** **a,b,** Simulated farfield radiation pattern from the cubic cavity (**a**) and electric field amplitude on [111] cut planes at different distances from the cube’s center (**b**) reveals the coexistence of 3D singularities. **c,** Experimentally measured masing emission spectrum at three starred directions in *x,y,z* away from singularities in (**a**). **d,** Experimentally measured normalized masing intensity on the loop *L* proves three coexisting masing zero-intensity singularities (Z1, Z2, Z3) as predicted in (**b**). Therefore, we confirm the existence of simultaneously masing and vortex associated singularities in three dimensions.


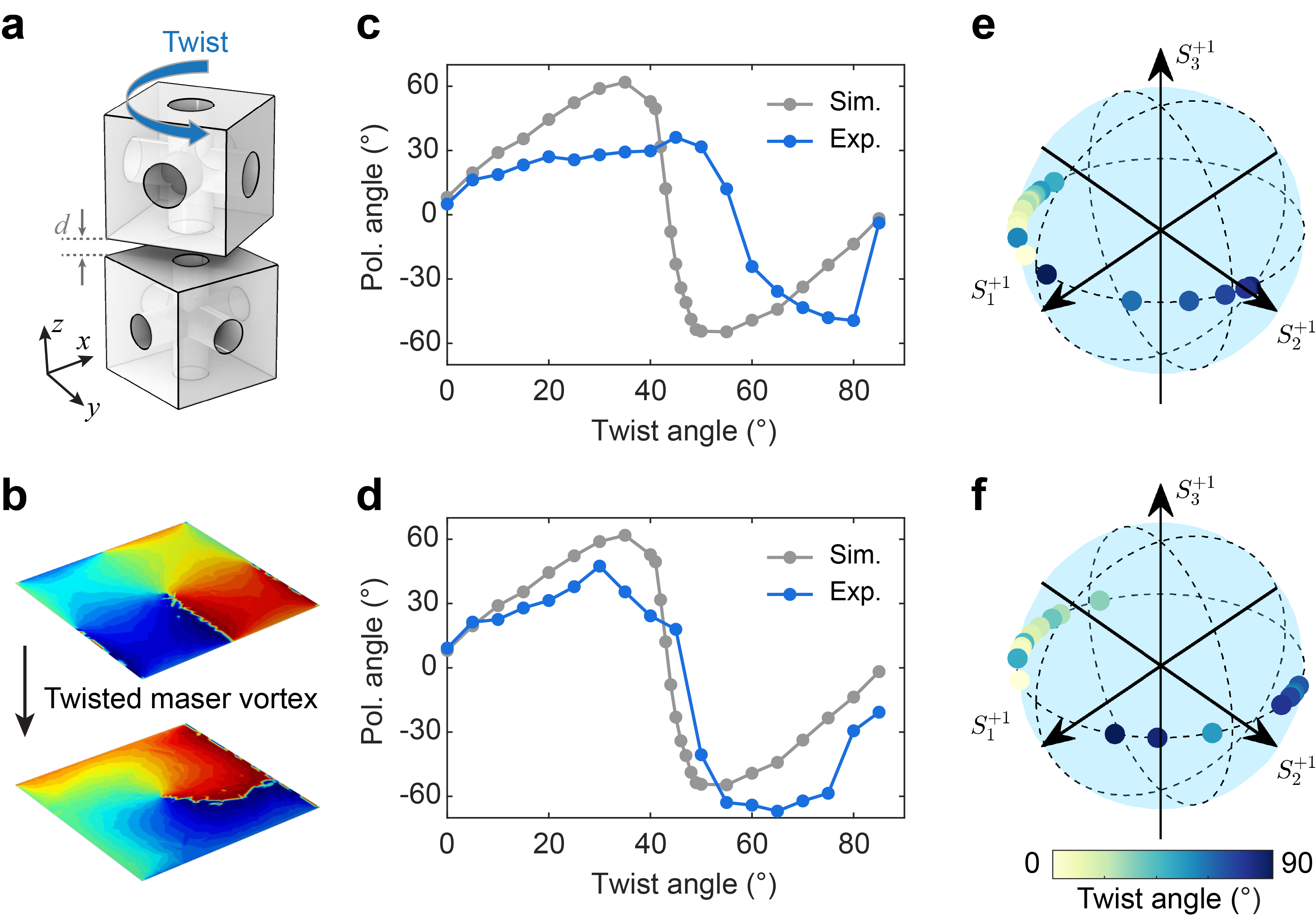


**Supplementary Fig. S11: Twisting masing vortex.** **a,** Schematic of two coupled cubic cavities with a relative twist angle that can be employed to twist polarization angle of masing vortex in (**b**). Simulated and experimentally measured polarization angle of masing vortex as a function of twist angle for two cubes with an air gap *d* of (**c**) 3 mm and (**d**) 6 mm. **e,f,** The corresponding measured masing vortex representation can be illustrated on higher-order Poincaré sphere. Elliptically and circularly polarized vortex emission with nonzero Stokes *S_3_* may be made possible with controllable phase delay between two cubes, for example, exciting gain mediums separately with phase-shifted optical delay lines.
